# A Second Opportunity for the Peptide-Based Analogues with γ-Lactam at the P1 Position: Human Cathepsin S Inhibition

**DOI:** 10.3390/ph18101462

**Published:** 2025-09-28

**Authors:** Santo Previti, Nunzio Iraci, Elsa Calcaterra, Roberta Ettari, Maria Zappalà

**Affiliations:** Department of Chemical, Biological, Pharmaceutical, and Environmental Sciences, University of Messina, Viale Ferdinando Stagno d’Alcontres 31, 98166 Messina, Italy; nunzio.iraci@unime.it (N.I.); elcalcaterra@unime.it (E.C.); rettari@unime.it (R.E.); mzappala@unime.it (M.Z.)

**Keywords:** cysteine proteases, Michael acceptors, docking studies, molecule repurposing, ketone warhead

## Abstract

**Background/Objectives**: SARS-CoV-2 pandemic led to the identification of peptide-based main protease (M^pro^) inhibitors. The overwhelming majority of them carry an electrophilic warhead and a γ-lactam at the P1 position. During the selectivity assessment of an *in-house* Michael acceptors targeting SARS-CoV-2 M^pro^, we unexpectedly observed a significant inhibition of human cathepsin S (hCatS). **Methods**: The biological investigation of three compounds (i.e., **SPR38**, **SPR39**, and **SPR41**) against hCatS was performed. The binding mode of SPRs was investigated by docking and molecular dynamics simulations. **Results**: Biological investigation has corroborated that hCatS is sensitive to peptide-based analogues harbouring γ-lactam at the P1 position and a vinyl methyl ketone warhead. In silico studies revealed that despite being solvent exposed, the γ-lactam at P1 might be involved in water-mediated H-bonds that could be optimized to gain inhibition potency and selectivity. **Conclusions**: The molecules repurposing of peptide-based SARS-CoV-2 M^pro^ inhibitors carrying the γ-lactam at the P1 site could pave the way for the identification of novel potent and selective hCatS ligands.

## 1. Introduction

Papain-like proteases are lysosomal proteolytic enzymes that have been identified in a variety of organisms, including animals, invertebrates, plants, and microorganisms [1]. In mammals, these types of proteases are commonly known as cathepsins. The human genome encodes for fifteen cathepsins, which can be classified as serine, aspartic, or cysteine proteases according to the amino acid responsible for the nucleophilic attack [2]. Cathepsins mediate a variety of physiological processes, including extracellular matrix degradation, intracellular protein turnover, neuronal cell homeostasis, and the processing of damaged proteins, immunoglobulins, and prohormones [3,4,5].

Human cathepsin S (hCatS or CTSS) is a cysteine protease biosynthesized as a pre-proenzyme of 331 amino acid residues [6]. The mature enzyme is constituted of a single-chain polypeptide comprising 217 amino acids and featuring the catalytic triad Cys25/His164/Asn184 [7]. hCatS is involved in multiple physiological and pathological processes, including inflammation [8], metabolic and neurological disorders [9], cancer [10], cardiovascular [11], renal [12], respiratory [13], and autoimmune diseases [14]. In addition to the lysosomal compartments, hCatS has also been detected extracellularly, where it has been shown to promote the degradation of extracellular proteins and angiogenesis [15]. Finally, this cysteine protease plays a pivotal role in regulating antigen processing and presentation through the major histocompatibility complex (MHC) II pathway in antigen-presenting cells (APCs) [16,17,18].

Given its involvement in various diseases, hCatS has emerged over the past decades as a promising therapeutic target and biomarker [19,20]. Several Structure–Activity Relationship (SAR) studies have resulted in the identification of interesting hCatS inhibitors [21,22,23,24,25], typically identified starting from compounds with potent inhibitory properties towards other human cysteine proteases and subsequently optimized through lead refinement strategies.

**Chart 1:**
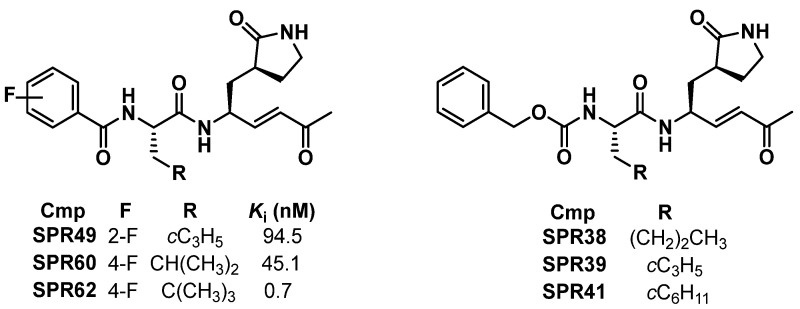
Structures and biological data towards hCatS of **SPR49**, **SPR60**, and **SPR62** [26]. Structures of **SPR38**, **SPR39**, and **SPR41** [30].

Recently, we reported the identification of dual SARS-CoV-2 M^pro^/human cathepsin L (hCatL) inhibitors [26]. Selectivity assays over a panel of human cysteine proteases revealed *K*_i_ values in the nanomolar and sub-nanomolar range towards hCatS for **SPR49**, **SPR60**, and **SPR62**, as illustrated in Chart 1. The three Michael acceptors carry a methyl vinyl ketone warhead, a γ-lactam at the P1, aliphatic amino acids at the P2 position, and a mono-fluorinated benzoyl ring as the *N*-cap. It is well established that the presence of Gln and its cyclic analogue (3*S*)-pyrrolid-2-one-3-yl-L-alanine adjacent to the cleavage site of SARS-CoV-2 M^pro^ was found to be essential for effective inhibition [27,28]. Since no known human proteases have the same cleavage specificity as SARS-CoV-2 M^pro^ [29], the such potent inhibition observed against hCatS was unexpected.

In light of these findings, we herein report the biological investigation of three *in-house* (3*S*)-pyrrolid-2-one-3-yl-L-alanine-containing Michael acceptors (i.e., **SPR38**, **SPR39**, and **SPR41**, shown in Chart 1) [30] towards hCatS, along with a discussion of SAR and molecular docking studies of the most promising inhibitors.

## 2. Results and Discussion

### 2.1. Biology

**SPR38**, **SPR39**, and **SPR41** were assayed against hCatS using Cbz-VVR-AMC as the fluorogenic substrate. All analogues exhibited potent inhibitory properties towards the target, with *K*_i_ values in the double-digit nanomolar range (Table 1). **SPR38** and **SPR39**, which feature norleucine (Nle) and cyclopropylalanine (Cpa), respectively, at the P2 site, showed comparable binding affinity. In contrast, the cyclohexylalanine (Cha)-derivative **SPR41** exhibited the most potent inhibitory effect (*K*_i_ = 10.7 ± 1.1 nM). The presence of Cha in **SPR41** resulted in 4- and 5-fold improvement in *K*_i_ values compared to Nle and Cpa in **SPR38** and **SPR39**, respectively. Compared to our previously reported SPR analogues targeting hCatS [26], only **SPR41**, with Cha at the P2 site, showed a *K*_i_ value in the low nanomolar range, similar to those observed for **SPR60** and **SPR62**.

The inhibitory properties of SPRs against hCatS were determined using *K*_i_ values, consistent with previously reported literature data [26,30]. Although both dialysis and dilution assays confirmed a covalent and irreversible binding mode of SPRs against SARS-CoV-2 M^pro^, hCatL, hCatB, and hCatS, time-dependent inhibition profiles did not exhibit sufficient curvature to allow accurate determination of the kinetic parameters *K*_i_, *k*_inact_, and *k*_2nd_. These findings support a bi-phasic inhibition mechanism, whereby high inhibitor concentrations promote irreversible inactivation of the enzyme, whereas at lower concentrations, the interaction remains predominantly reversible.

The three analogues showed notable selectivity towards hCatS. In fact, inhibitory properties in the micromolar range towards hCatL and hCatB were observed, except for **SPR41**, for which submicromolar activity against hCatL was determined. This trend highlights the contribution of the common binding region of the SPRs, namely the methyl vinyl ketone warhead, a γ-lactam residue and an aliphatic amino acid at the P1 and P2, respectively, provided for strong hCatS inhibition with *K*_i_ values in the low nanomolar or even subnanomolar range. Conversely, hCatL and hCatB appear to be less sensitive to SPRs. Except for the Leu-containing derivative **SPR60**, the other Michael acceptors displayed only moderate activity towards hCatL, with *K*_i_ values in the submicromolar and micromolar range. For hCatB, even higher values of *K*_i_ were observed, resulting in selectivity indices ranging from 157 to 11 000. These findings indicate that SPRs are highly compatible with the catalytic site of hCatS, while exhibiting markedly reduced affinity for hCatL and, in particular, hCatB.

**Chart 2:**
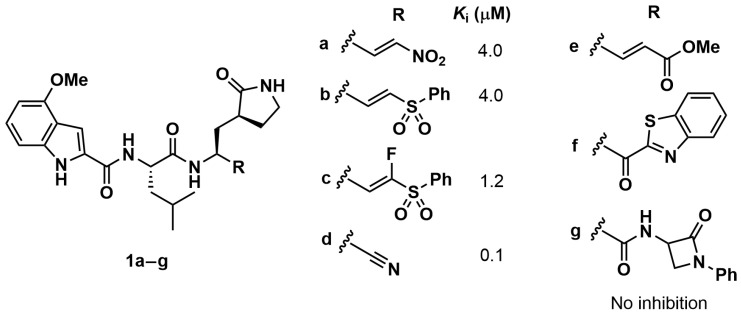
Structures and biological activity of compounds **1a–g** towards hCatS, previously reported by Schirmeister and co-workers [32].

To the best of our knowledge, no hCatS inhibitors incorporating (3*S*)-pyrrolid-2-one-3-yl-L-alanine have been rationally developed to date. However, Schirmeister and co-workers assayed a series of peptide-based compounds bearing a Gln pentatomic surrogate at the P1 position towards hCatS (Chart 2) [32]. Analogues **1a**–**d** showed *K*_i_ values in the micromolar and submicromolar range, whereas derivatives **1e**–**g** showed no measurable inhibitory activity. Since these compounds differ exclusively in the warhead, it is evident that this moiety plays a crucial role for hCatS inhibition. Specifically, the nitro and sulfonyl phenyl moieties of Michael acceptors **1a** and **1b**–**c**, respectively, as well as the nitrile warhead of **1d**, were found to be suitable for hCatS inhibition, whereas, the presence of vinyl methyl ester (**1e**), benzothiazole (**1f**), and β-lactam ring (**1g**) warheads were ineffective. Based on these findings reported in the literature and our results with SPR analogues, the methyl vinyl ketone warhead appears highly suitable for hCatS inhibition, regardless of surrounding binding regions. Notably, Leu derivative **SPR60** showed a single-digit *K*_i_ value in the nanomolar range, representing a two/three-orders-of-magnitude improvement over Leu-analogues **1a**–**d**. Moreover, the presence of the further branched amino acid (i.e., *tert*-butylalanine, known as Tba, in **SPR62**) enhanced hCatS affinity and conferred remarkable selectivity over hCatL (SI = 1000) and hCatB (SI = 11 000).

**Chart 3:**
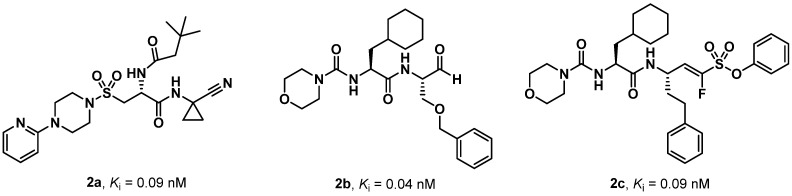
Structures and biological activity of rationally designed hCatS inhibitors known in the literature: **2a** [22], **2b** [23], and **2c** [33].

As previously mentioned, several potent hCatS inhibitors have been rationally developed. Here, we compare some of these reference compounds with the most active SPR analogues, namely **SPR41**, **SPR60**, and **SPR62**. The vinyl sulfone **K11017** (Mu-Leu-hPh-VSPh), also known as LHVS, is widely employed as a positive control in hCatS inhibition assays [31]. **SPR41** and **SPR60** showed binding affinities comparable to **K11017**, whereas **SPR62** exhibited a *K*_i_ value approximately one order of magnitude lower. A similar trend was observed when comparing SPRs with **BI-1124** and **BI-1195**, two peptidomimetics carrying the nitrile warhead developed by Boehringer Ingelheim. Both compounds are used as reference inhibitors of hCatS, with a *K*_d_ value of 9 and 31 nM. Notably, **SPR62**, the most potent and selective SPRs (*K*_i_ = 0.7 nM), exhibited a *K*_i_ value only one order of magnitude higher compared to compounds **2a–c** (Chart 3) [22,23,33], which represent some of the most promising hCatS inhibitors rationally developed in three different SAR studies and known in the literature.

During the SARS-CoV-2 pandemic, drug repurposing emerged as a key strategy for the rapid development of effective antiviral agents [34,35]. The SPR analogues discussed herein was originally developed as potential SARS-CoV-2 M^pro^ inhibitors, with their hCatS inhibitory properties identified only during selectivity profiling. This observation suggests that further promising hCatS inhibitors could be discovered among compounds initially developed for SARS-CoV-2 M^pro^. Indeed, an impressive number of SAR studies have reported the development of peptide-based SARS-CoV-2 M^pro^ inhibitors carrying an electrophilic warhead, the (3*S*)-pyrrolid-2-one-3-yl-L-alanine residue at the P1 site, an aliphatic amino acid at the P2 position, and with further optimization targeting the S3, S4, and S5 subsites [36]. The biological investigation of these molecules towards hCatS could provide valuable insights and enable the identification of novel building blocks and warheads useful for the hCatS inhibition. In fact, a wide range of warheads have been successfully investigated for SARS-CoV-2 M^pro^ inhibition, including vinyl sulfonamide, aldehyde, nitrile, Michael acceptors, ketoamide, 4-chlorobut-2-ynamide, hydroxymethylketone, and benzyloxymethylketone, amongst others [37,38,39]. While nitriles, Michael acceptors, and aldehydes, have been studied for hCatS inhibition, the potential of diverse reactive warheads remains largely unexplored. Furthermore, the nature of the electron-withdrawing groups (EWGs) in Michael acceptors significantly influences hCatS inhibitory potency, as evidenced by comparison among **SPR62**, **K11017**, **1a–c**, **1e**, and **2a**. Before the SARS-CoV-2 pandemic, the incorporation of γ-lactam at the P1 site of peptidomimetics was restricted to the development of antiviral agents. Considered the promising results obtained with SPRs, (3*S*)-pyrrolid-2-one-3-yl-L-alanine-containing compounds could be rationally developed for hCatS inhibition. Drug/molecule repurposing of compounds originally designed as SARS-CoV-2 M^pro^ inhibitors could therefore accelerate the identification of promising warheads, building blocks, and lead compounds for hCatS inhibition.

### 2.2. Docking Studies

**Figure 1:**
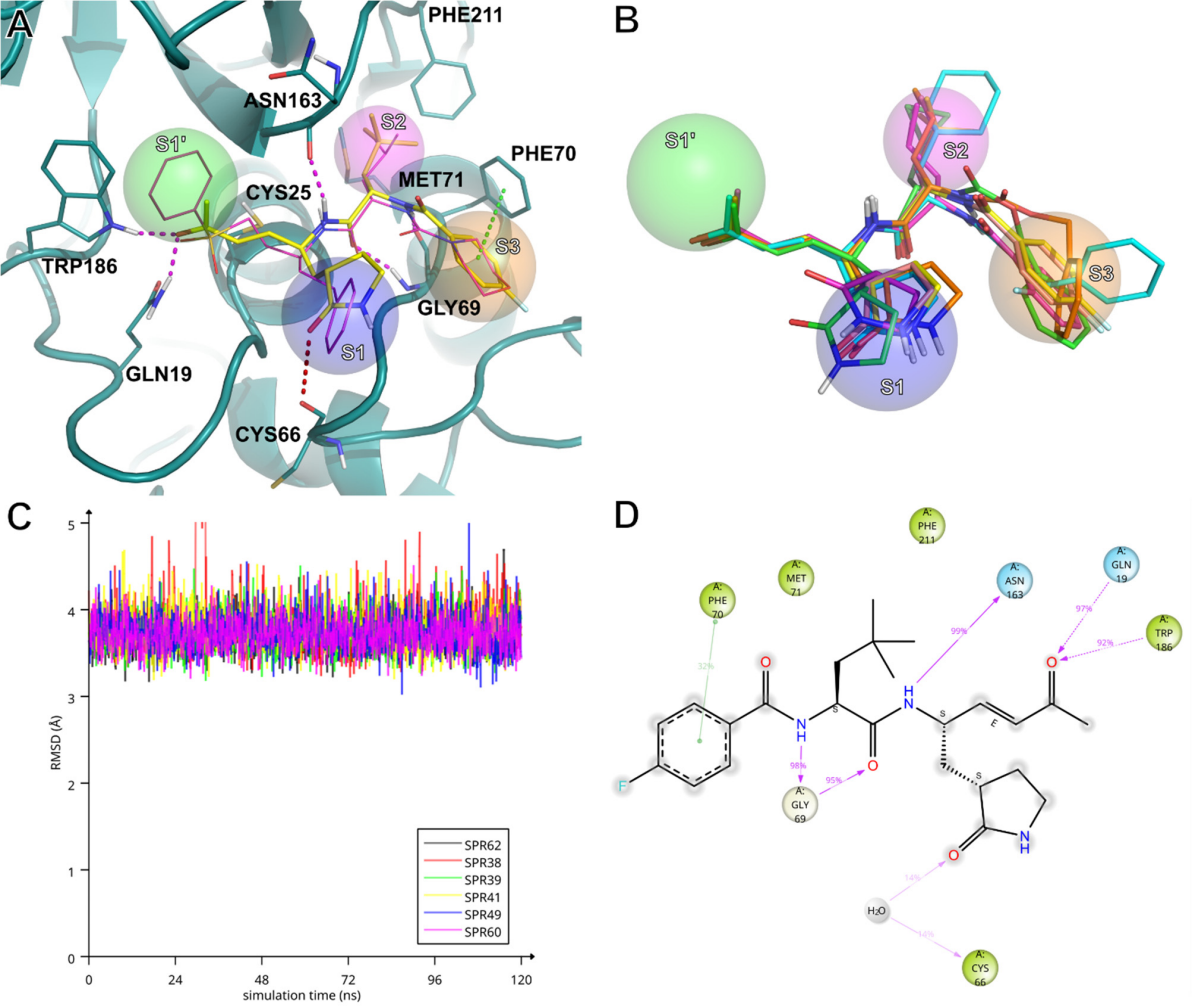
(**A**) Docking-predicted bound conformation of **SPR62** (yellow sticks), into hCatS (teal cartoons and sticks). H-bond interactions are highlighted by magenta dashed lines, π-π stacking interactions by green dashed lines. The experimentally solved bound conformation of **K11017** (magenta thin sticks) is shown as reference [41]. (**B**) Docking-predicted hCatS-bound conformations of **SPR38** (orange sticks), **SPR39** (green sticks), **SPR41** (cyan sticks), **SPR49** (magenta sticks), **SPR60** (salmon sticks), **SPR62** (yellow sticks). (**C**) Distance between the Cys 25 γ-sulphur and the warhead β-carbons of inhibitors. (**D**) Simulation interactions diagram of **SPR62** in complex with hCatS. Only residues interacting with the ligand for at least 12 out of 120 ns of MD simulation are shown. Residues are coloured according to the following scheme: cyan, polar; green, hydrophobic; grey, water molecule. Grey halos highlight solvent exposure. H-bonds are represented by magenta arrows (dashed when side-chain atoms are involved, solid in the case of backbone atoms involvement); green solid lines represent π−π stacking interactions.

**Figure 2:**
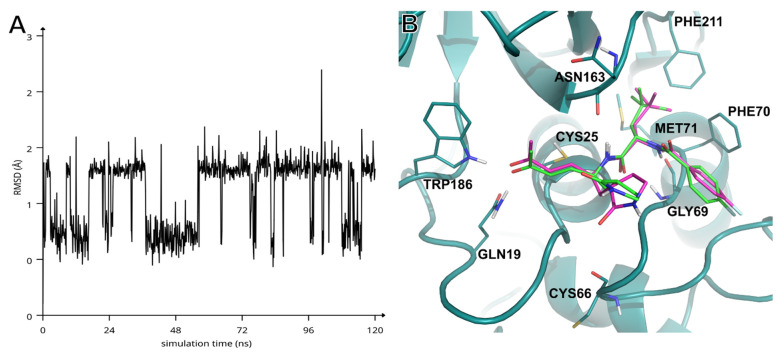
(**A**) RMSD values of **SPR62** atoms as a function of MD simulation time. (**B**) Representative structures of the “up” cluster (green sticks) and of the “down” cluster (magenta sticks). hCatS is depicted in teal cartoons and sticks.

Compounds **SPR38**, **SPR39**, **SPR41**, **SPR49**, **SPR60**, and **SPR62** were investigated in silico for their molecular recognition at the active site of hCatS using the extended sampling protocol of the Schrödinger Induced Fit Docking (IFD) tool [40]. As expected, docking results confirmed that the P1, P2, and P3 residues appropriately occupy the S1, S2 and S3 sub-pockets of hCatS (Figure 1A,B—IFD poses can be found in Supplementary Materials, in PDB format). It was observed that the β-carbon atoms of the inhibitors’ warhead stably positioned in close proximity to the γ-sulphur of the catalytic Cys25 throughout 120 ns-long molecular dynamics (MD) simulations (Figure 1C). Overall, all SPRs showed highly similar interaction patterns throughout the MD simulations (Figure S1). Several studies have addressed which face is involved in the nucleophilic attack and, thereby, the configuration of the new chirality centre at the sp3-hybridized carbon [41,42,43]. In our case, it was predicted that the nucleophilic attack of the Cys25 thiolate occurs from the re face of the inhibitors. Consequently, the pivotal reaction between the Cys25 thiolate and prochiral electrophilic β-carbon would result in the formation of an (*S*)-chirality centre. Figure 1C depicts the key interactions between hCatS and **SPR62** during the MD simulation, highlighting stable contacts with multiple residues within the active site. At the P2 position, Tba residue of **SPR62** forms strong hydrogen bonds with the backbone of Gly69 through both its NH and carbonyl oxygen. The P1 NH group of **SPR62** acts as a hydrogen bond donor, interacting with the backbone carbonyl of Asn163. The warhead’s carbonyl oxygen accepts two additional hydrogen bonds from the side chain NH groups of Gln19 and Trp186. Futhrer stabilizing interactions include π–π stacking with Phe70 and hydrophobic contacts with Met71 and Phe211. MD trajectory analysis revealed that the γ-lactam moiety, although solvent-exposed and lacking direct residue contacts, occasionally engages in a water-mediated hydrogen bond between its carbonyl oxygen and that of Cys66. At the same time, ligand’s root mean square deviation (RMSD) analysis indicates that **SPR62** is plausibly oscillating between two bound conformations over the course of the simulation (Figure 2A). Notably, the γ-lactam carbonyl oxygen exhibits the highest root mean square fluctuation (RMSF) value among all atoms of **SPR62** (1.863 Å), supporting the hypothesis that the conformational variability arises mainly from alternative orientations of the γ-lactam ring.

To further explore this behaviour, we performed cluster analysis on the trajectory (see Supplementary Materials), which identified two significantly populated conformational clusters (Table S1). The clusters contained **SPR62** mainly differing for the orientation of the γ-lactam carbonyl group: either pointing “up” (Figure 2B, yellow sticks, cluster 1) or “down” toward the carbonyl of Cys66 (Figure 2B, magenta sticks, cluster 2). Single-point energy calculations (in vacuum) averaged over all ligand conformations within each cluster revealed that the “down” conformation carries an energetic penalty of approximately 3 kcal/mol (Table S1). However, this cost may be rewarded by water-mediated hydrogen bonds involving Cys66 or neighbours such as Lys64 or Asn67 (Figure S1).

Despite this energy difference, both the two conformational clusters are significantly populated (539 vs. 409), suggesting a dynamic equilibrium. This observation warrants future investigation into the role of structured water molecules near Cys66 and their potential exploitation to enhance potency and selectivity toward hCatS.

## 3. Materials and Methods

### 3.1. Biological

The biological assays against hCatS were performed in accordance with the methods previously reported by Prof. Schirmeister’s research group [23,33]. Briefly, the inhibitory activities of **SPR38**, **SPR39**, and **SPR41** against hCatS (purchased form Merck, Darmstadt, Germany) were evaluated using the fluorogenic substrate Cbz-Val-Val-Arg-AMC (Bachem, Bubendorf, Switzerland). The fluorescence was recorded in white flat-bottom 96-well plates (Greiner Bio-One, Kremsmünster, Austria) with a Tecan Spark 10M plate reader (Tecan Group Ltd., Männedorf, Switzerland). Stock solutions of the substrate and SPRs were prepared in DMSO. Each well contained 200 µL, consisting of 180 µL assay buffer, 5 µL enzyme solution in buffer, 10 µL inhibitor in DMSO (or DMSO alone as negative control), and 5 µL substrate. Assay buffer consisted of 50 mM potassium phosphate, 2.5 mM DTT, and 2.5 mM EDTA at pH 6.5, while the enzyme buffer consisted of 35 mM potassium phosphate, 35 mM sodium acetate, 2 mM DTT, and 2 mM EDTA at pH 6.5. All chemicals and reagents used for enzyme and assays buffer, along with DMSO, were purchased from Merck (Darmstadt, Germany). The enzyme (10 nM) was diluted and pre-incubated in enzyme buffer at room temperature for 20 min before trnsfer to the assay buffer. The reaction was initiated by adding the substrate (10 µM) under rigorous mixing. The reaction was monitored for 10 min at 25 °C, and fluorescence readout was performed in 30 sec intervals. AMC release was detected using an excitation wavelength of 380 nm and an emission wavelength of 460 nm. Residual enzymatic activity was calculated from the substrate hydrolysis rate in the presence of inhibitors relative to the DMSO control. To determine the IC_50_ values dilution series of the inhibitors were prepared (eg. final concentrations: 100, 30, 10, 3, 1, 0.3, 0.1 μM, and DMSO as control). The IC_50_ values were calculated using GRAFIT [44] by fitting the residual enzymatic activity to the four-parameter IC_50_ equation (Equation (1)):

(1) $$Y=\frac{Y_{max}-Y_{min}}{1+{\left(\frac{\left[I\right]}{{IC}_{50}}\right)}^{s}}$$

where *Y* is the substrate hydrolysis rate, *Y_max_* is the maximum value of the dose response curve at inhibitor concentrations [*I*] = 0 µM, *Y_min_* is the minimum value at high inhibitor concentrations, and s is the Hill coefficient [45].

To calculate the *K*_i_ value, Equation (2) was used:

(2) $$K_{i}=\;\frac{{IC}_{50}}{1+\frac{\left[S\right]}{K_{m}}}$$

with *K*_m_ as the substrate concentration at which the hydrolysis activity is half maximal and [*S*] as the substrate concentration [45].

### 3.2. Molecular Modelling

#### 3.2.1. Target Structure Preparation

The experimental structure of hCatS in complex with **K11017** (PDB ID: 1NPZ [41]) was obtained from the RCSB Protein Data bank [46]. The covalently bound inhibitor was initially removed, and the structure was processed using the Schrodinger Protein Preparation workflow to optimize the target structure for the following studies [47]. Following previous protocols [48], hydrogen atoms were added and bond orders were assigned. The hydrogen-bonding network was optimized by reorienting hydroxyl and thiol groups, the amide groups of Asn and Gln, and imidazole rings of His residues. The ionization and tautomeric states of His, Asp, Glu, Arg, and Lys residues were adjusted to match physiological pH of 7.4 using PROPKA. The structure was then submitted to a minimization (OPLS2005 force field [49]) that was stopped when RMSD of heavy atoms reached 0.30 Å. All crystallographic water molecules were removed prior to further analysis.

#### 3.2.2. Preparation of Ligands

Ligands were sketched using the Maestro GUI v2025-2 [50] and were then submitted to a conformational search using MacroModel v2025-2 [51]. A Low-Mode Conformational Search (LMCS) method was performed, with a maximum of 1000 steps per structure. Calculations were carried out in implicit solvent (GB/SA) and energy minimization was performed on each conformer using the PRCG method, with a maximum of 2500 iterations per step. For each ligand, only the global minimum energy conformation was retained for subsequent docking simulations.

#### 3.2.3. Molecular Docking Simulations

The Schrödinger Induced Fit Docking (IFD) v2025-2 workflow was used to explore the binding mode of the ligands [40]. Residues within 5 Å of the experimentally bound conformation of **K11017** were selected as flexible. In the initial docking stage, the docking space was centred on the crystallographic pose of **K11017**, and defined as a (30 Å)^3^ cubic box, with a smaller, nested, (15 Å)^3^ cubic one where the diameter midpoint of each docked ligand was required to stay. After the Prime refinement of flexible residues [52], the second docking stage (redocking) was performed assigning the outerbox size automatically, based on the docking pose from the first docking stage. Both first docking and redocking were performed using GlideSP v2025-2 [53]. Final docking poses were scored and ranked using the default IFDscore, the best scoring complex for each ligand was used as input for the molecular dynamics simulations.

#### 3.2.4. Molecular Dynamics Simulations

MD simulations were set and run using Desmond v2025-2 [54]. Simulated environments were built using the TIP3P water model [55] and the OPLS 2005 force field [49]. Prior to the 120 ns-long production stage, the systems were relaxed using the default multistep protocol. After system relaxation, 120 ns-long MD simulations were carried out at a 300 K in the NPT ensemble using a Nose-Hoover chain thermostat and a Martyna-Tobias-Klein barostat (1.01325 bar), applying a 1 kcal/mol harmonic constrain on the backbone heavy atoms. Time steps were set to 2 fs, 2 fs and 6 fs for bonded, near, and far interactions, respectively. Structures were sampled every 120 ps and trajectories were analyzed using Maestro [50] and VMD [56].

#### 3.2.5. Trajectory Clustering and Energy Evaluation of Conformers

Snapshots of the **SPR62**/hCatS complex conformations were extracted from the MD trajectory. Conformations were superimposed by the backbone heavy atoms of hCatS and then **SPR62** bound conformations were clustered using the conformer_cluster.py script [57], based on atomic RMSD values. RMSD calculations were performed in place (using the previous hCatS backbone alignment) on the amide motif (excluding the hydrogen atom) of the γ-lactam. Merge distance cutoff was set to 2 Å, using the average linkage clustering method. **SPR62** conformations were evaluated for their ligand energies (in vacuum), and the energies of each conformation were averaged for each of the two most populated clusters.

## 4. Conclusions

Molecules originally developed as SARS-CoV-2 M^pro^ inhibitors showed potent affinity towards hCatS. These compounds carry a methyl vinyl ketone warhead, a γ-lactam Gln derivative at the P1 site, different aliphatic amino acids at the P2 site, and either a fluorine-containing phenyl ring or Cbz as the *N*-capping. Herein, **SPR38**, **SPR39**, and **SPR41**, differing only in their P2 residue, showed a relevant double-digit nanomolar affinity for hCatS, along with selectivity indices ranging from 24 to 74 over hCatL and from 175 to 1354 and hCatB. The biological data herein reported confirm the significant inhibitory properties against hCatS of peptide-based Michael acceptors originally developed for SARS-CoV-2 M^pro^. All the SPR derivatives share the P1-warhead fragment, namely the (3*S*)-pyrrolid-2-one-3-yl-L-alanine at the P1 site and a methyl vinyl ketone warhead. Comparison with known hCatS inhibitors highlighted this fragment as a key determinant for hCatS inhibition. Molecular modelling studies provided insight into the role of the solvent-exposed P1 γ-lactam substituent in target recognition. Furthermore, in silico analyses also suggested two additional strategies for the potential optimization of potency and selectivity toward hCatS, such as *i*) the design of modified P1 substituents capable to make more efficient water mediated H-bonds, or *ii*) the introduction of extended substituents able to displace structural water molecules and establish direct interactions with residues such as Cys66. Overall, peptide-based Michael acceptors, originally developed for SARS-CoV-2 M^pro^, demonstrated potent and selective inhibition of hCatS. Since a large number of these compounds carries a γ-lactam Gln derivative at the P1 and an electrophilic warhead, the repurposing strategy of molecules with this fragment could provide significant data for hCatS inhibition.

## Figures and Tables

**Table 1:** Biological data for **SPR38**, **SPR39**, and **SPR41** and comparison with known (3*S*)-pyrrolid-2-one-3-yl-L-alanine-containing analogues and **K11017** (positive control).

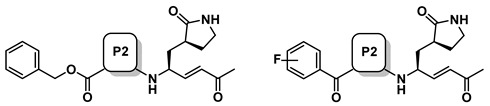							
Compounds	P2	F	*K*_i_ (nM)			SI hCatL/hCatS	SI hCatB/hCatS
			hCatS	hCatL	hCatB		
**SPR38**	Nle	-	58.8 ± 3.6	1920 ± 100 ^b^	11,100 ± 1200 ^b^	32	189
**SPR39**	Cpa	-	45.1 ± 3.8	3380 ± 220 ^b^	7880 ± 650 ^b^	75	175
**SPR41**	Cha	-	10.7 ± 1.1	252 ± 18 ^b^	14,400 ± 1200 ^b^	24	1345
**SPR49 ^a^**	Cpa	2-F	95.4 ± 0.6	294 ± 42	>15,000	3	>157
**SPR60 ^a^**	Leu	4-F	6.23 ± 0.25	20 ± 2	10,900 ± 500	3	1750
**SPR62 ^a^**	Tba	4-F	0.7 ± 0.1	701 ± 66	7700 ± 320	1000	11,000
**K11017 ^c^**	-	-	5.9				

^a^ data from [26]; ^b^ data from [30]; ^c^ data from [31].

## Data Availability

Data presented in this study is contained within the article and Supplementary Material. Further inquiries can be directed to the corresponding authors.

## Supplementary Materials

The following supporting information can be downloaded at https://www.mdpi.com/article/10.3390/ph18101462/s1: Figure S1: Protein-Ligand Contacts of hCatS bound to SPR38, SPR39, SPR41, SPR49, SPR60, and SPR62. Table S1. Clustering statistics and energetics of SPR62 conformations from the MD simulation. SPR38.pdb: Docking-predicted SPR38/hCatS complex. SPR39.pdb: Docking-predicted SPR39/hCatS complex. SPR41.pdb: Docking-predicted SPR41/hCatS complex. SPR49.pdb: Docking-predicted SPR49/hCatS complex. SPR60.pdb: Docking-predicted SPR60/hCatS complex. SPR62.pdb: Docking-predicted SPR62/hCatS complex.

## Abbreviations

The following abbreviations are used in this manuscript:

AMC	7-amino-4-methyl coumarin
APC	Antigen-Presenting Cell
Cbz	Carbobenzyloxy
Cha	Cyclohexylalanine
Cpa	Cyclopropylalanine
EWG	Electron-Withdrawing Group
hCatB	Human Cathepsin B
hCatL	Human Cathepsin L
hCatS	Human cathepsin S
IFD	Induced Fit Docking
LMCS	Low-Mode Conformational Search
MD	Molecular Dynamics
MHC	Major Histocompatibility Complex
M^pro^	Main protease
Nle	Nor-leucine
PDB	Protein Data Bank
RMSD	Root Mean Square Deviation
RMSF	Root Mean Square Fluctuation
SAR	Structure-Activity Relationship
SARS-CoV-2	Severe Acute Respiratory Syndrome Coronavirus 2
