# Supplementary material for: A Second Opportunity for the Peptide-Based Analogues with γ-Lactam at the P1 Position: Human Cathepsin S Inhibition
Source: Pharmaceuticals (Basel). 2025 Sep 28;18(10):1462. doi: 10.3390/ph18101462 (PMC12566624; doi:10.3390/ph18101462)
Supplement: Supplementary file 1 [file pharmaceuticals-18-01462-s001.zip › pharmaceuticals-3894905-supplementary.pdf]

SUPPLEMENTARY MATERIALS

A Second Opportunity for the Peptide-Based Analogues with γ-Lactam at the P1 Position: Human Cathepsin S Inhibition

Santo Previti *, Nunzio Iraci, Elsa Calcaterra, Roberta Ettari and Maria Zappalà

Department of Chemical, Biological, Pharmaceutical, and Environmental Sciences, University of Messina, Viale Ferdinando Stagno d'Alcontres 31, 98166 Messina, Italy; nunzio.iraci@unime.it (N.I.); elcalcaterra@unime.it (E.C.); rettari@unime.it (R.E.); mzappala@unime.it (M.Z.)

* Correspondence: spreviti@unime.it; Tel.: +39-090-6765669

INDEX

Figure S1……………………………………………………………………………………………..S1

Table S1………………………………………………………………………………………………S2

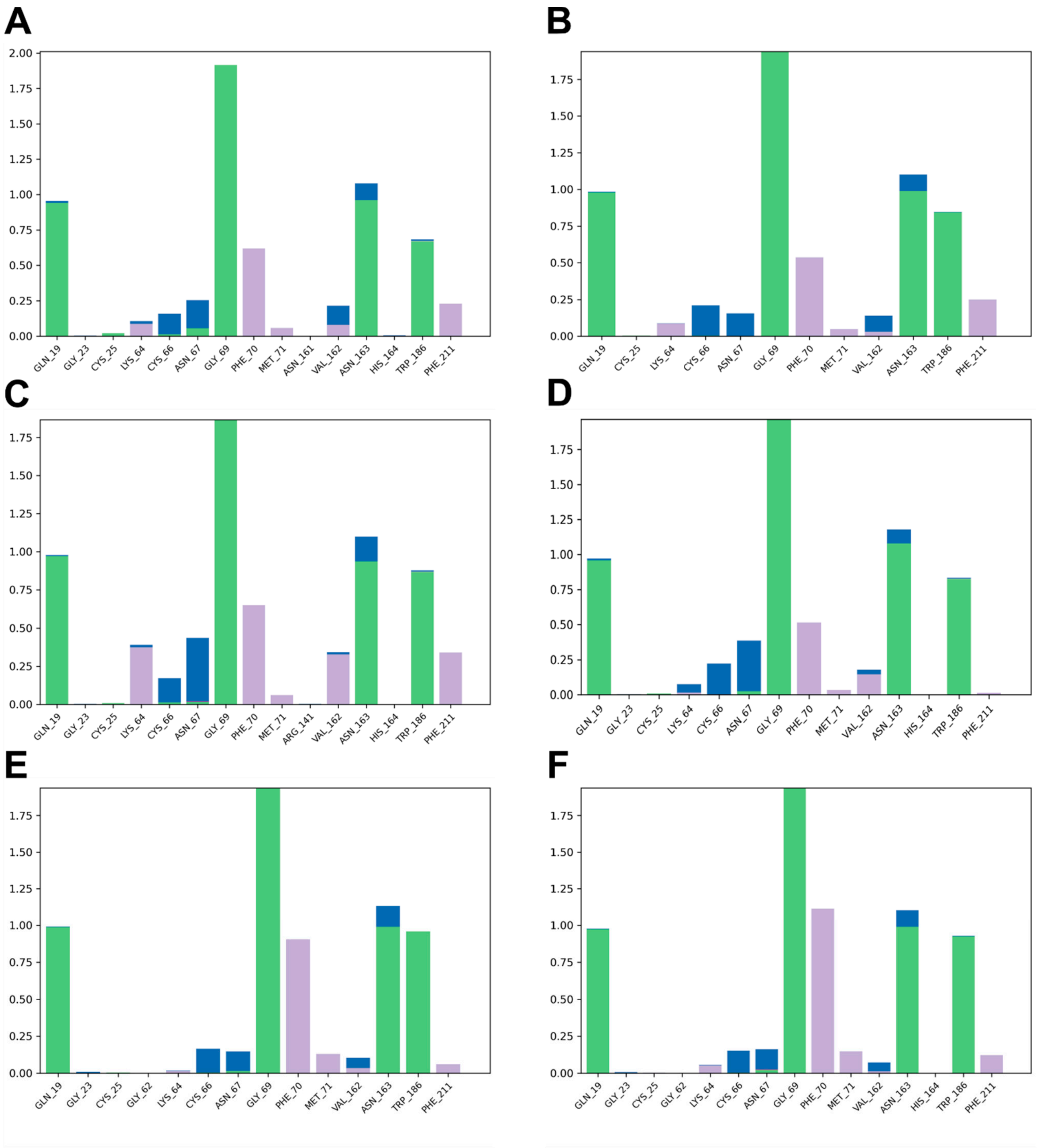

Figure S1. Protein-Ligand Contacts of hCatS bound to **SPR38** (A), **SPR39** (B), **SPR41** (C), **SPR49** (D), **SPR60** (E), and **SPR62** (F). The stacked bars are normalized over the simulation time. Bars are color-coded by interaction, as follows: H-bonds – green; Hydrophobic – light purple; Water bridges – blue.

Table S1. Clustering statistics and energetics of **SPR62** conformations from the MD simulation.

Cluster rank (by population)	Cluster size	Cluster variance	Average ligand energy (*in vacuum*)
1	539	1.708 Å^2	4.55 kcal/mol
2	409	0.399 Å^2	7.51 kcal/mol
3	36	1.311 Å^2	-*
4	12	0.296 Å^2	-*
5	2	0.615 Å^2	-*
* not calculated.			
